# Brazilian registry for the elimination of hepatitis C in dialysis units: a call to action for Nephrology

**DOI:** 10.1590/2175-8239-JBN-2021-0050

**Published:** 2021-07-07

**Authors:** José A. Moura-Neto, Maria Lúcia Gomes Ferraz, Paulo Lisboa Bittencourt, Osvaldo Merege Vieira

**Affiliations:** 1 Sociedade Brasileira de Nefrologia, São Paulo, SP, Brasil.; 2 Sociedade Brasileira de Hepatologia, São Paulo, SP, Brasil.; 3 Instituto Brasileiro do Fígado, São Paulo, SP, Brasil.

**Keywords:** Dialysis, Kidney Dialysis, Hepatitis C, Chronic Hepatitis, Hepacivirus, Hepatitis, Viral, Human, Disease Eradication, Diálise, Diálise Renal, Hepatite C, Hepatite Crônica, Hepacivirus, Hepatite Viral Humana, Erradicação de Doenças

## Abstract

Infection by the hepatitis C virus is more prevalent in patients on dialysis than in the general population in Brazil, and has been associated with worse outcomes. Current therapy for hepatitis C is highly effective, safe, and widely available in Brazil, with coverage provided to dialysis patients with chronic kidney disease, which makes the elimination of hepatitis C a viable target. The Brazilian Society of Nephrology, the Brazilian Society of Hepatology, and the Brazilian Liver Institute developed the “Brazilian Registry for the Elimination of Hepatitis C in Dialysis Units”. This project aims to identify, treat, and monitor the response to treatment of patients on chronic dialysis infected with the hepatitis C virus in Brazil. This article presents the issue and invites Brazilian nephrologists to rally around the achievement of a significant goal.

## Resumo

A infecção pelo vírus da hepatite C é mais prevalente em pacientes em diálise do que na população geral no Brasil e implica um pior prognóstico. O tratamento atual para hepatite C é altamente eficaz, seguro e disponível no país, inclusive para a população de pacientes crônicos em diálise, o que torna a eliminação do vírus da hepatite C uma meta viável. A Sociedade Brasileira de Nefrologia, a Sociedade Brasileira de Hepatologia e o Instituto Brasileiro do Fígado desenvolveram o “Registro Brasileiro para Eliminação da Hepatite C nas Unidades de Diálise”. O projeto visa identificar pacientes em diálise crônica com vírus da hepatite C no Brasil, além de tratar e monitorar a resposta virológica após o tratamento. Este breve artigo apresenta o problema e convida os nefrologistas brasileiros a unirem forças nesse objetivo comum.

Hepatitis C is a relevant cause of liver disease and a severe global health issue. Although the virus was isolated in 1989^1^ - until then the disease was called non-A, non-B hepatitis -, hepatitis C recently gained international attention. In October 2020, three physicians won the Nobel Prize in Physiology or Medicine for research on the hepatitis C virus (HCV)^2^.

The prevalence of HCV infection in the world is approximately 1%, with significant differences between regions. More than 70 million individuals live with hepatitis C globally, a condition known for increasing the risk for hepatocellular carcinoma and liver cirrhosis. In 2015, 1.34 million people died of hepatitis C - a number greater than that of deaths by HIV globally. Although current therapy cures more than 95% of the individuals with hepatitis C, the World Health Organization recognizes that access to diagnostic tests and adequate treatment is still insufficient^3^.

Among individuals on dialysis, prevalence tends to be much higher than in the general population. Data from the 2020 Dialysis Census organized by the Brazilian Society of Nephrology indicate that 2.8% of the patients on dialysis in the nation are positive for HCV^4^. This means that approximately 4,000 of an estimated population of individuals on dialysis of 144,795 are infected with HCV. For purposes of comparison, the prevalence of HCV in dialysis units was 4.2% in 2013^5^. Although the numbers are trending down, a sharper decline was expected on account of the existence and availability of treatment. The prevalence of hepatitis B virus infection, for example, dropped from 1.4% to 0.7% within the same time period^4^^,^ 5. In fact, patients with HCV are undertreated in dialysis units^6^. Logistical issues, ranging from access to tests and medication, cost and little information on the matter, might be linked to low treatment penetration rates. Furthermore, cases might by underreported, since fewer than 30% of the renal replacement therapy units in Brazil voluntarily participated in the latest Dialysis Census survey. Little participation and engagement from dialysis units might cause selection bias (participation or non-response bias), which indicate that the situation might be potentially worse. A study reported positive serology tests in 6.5% of the patients^7^.

Transmitted through percutaneous exposure to blood and blood products or organs transplanted from a previously infected donor, hepatitis C viremia places seronegative patients receiving treatment on the same facility and healthcare workers at risk. Patients on dialysis have higher death rates than the general population. Additionally, positive HCV serology in individuals on maintenance hemodialysis has been associated with significantly more deaths by cardiovascular causes^8^.

Since it is a disease of parenteral transmission via percutaneous exposure to contaminated blood or blood products, hepatitis C poses risk to healthcare workers with the possibility of contagion to patients treated at dialysis units.

On account of the complex measures involved in the elimination of HCV, in 2017 the European Association for the Study of the Liver recommended that the global goal should be broken down into smaller targets. This public policy strategy to eliminate HCV in the population is known as “micro-elimination”^9^, with dialysis units targeted as one of the intervention subgroups^10^.

**Figure 1:**
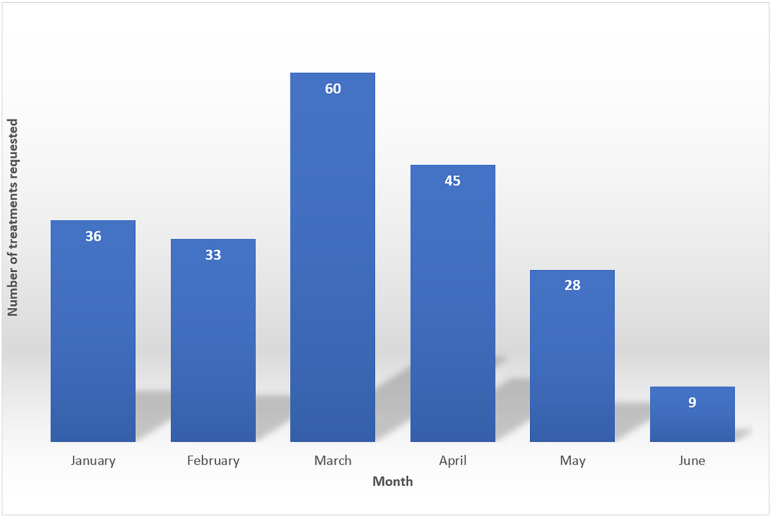
Treatment requests submitted to the Ministry of Health for patients with hepatitis C and advanced chronic kidney disease (glomerular filtration rate < 30mL/min/1.72m^2^) between January and June 2020^10^.

Since there is no vaccine against hepatitis C, prevention and treatment constitute the bases for public health policy devised to eliminate HCV. Glecaprevir/Pibrentasvir, a combination of drugs against multiple HCV genotypes, was recently described as safe and effective for patients with kidney disease^11^. The low chance of side effects and the few drug interactions reported for the drug combination make it a good candidate for use in populations on dialysis, which led to its incorporation in the treatment protocol available through the Brazilian Public Healthcare System. The Clinical Protocol and Therapeutic Guidelines for hepatitis C and co-infections approved in Ordinance 84 of December 19, 2018, and Informative Note 13/2019, guide the treatment of individuals in this group. The recommendation states that patients with advanced chronic kidney disease with an estimated glomerular filtration rate (eGFR) below 30mL/min/1.72m^2^ should take three tablets of Glecaprevir/Pibrentasvir 100 mg/40 mg orally once a day. Treatment lasts eight weeks for patients without liver cirrhosis and twelve weeks for subjects with Child class A cirrhosis. Although the medication has been available in the Brazilian Public Healthcare System since 2019, the issue is far from resolved. In the first half of 2020, only 211 prescriptions were requested from the Ministry of Health - the equivalent to less than 10% of the population on dialysis estimated to have HCV infection in Brazil in the time period (Figure 1)^10^. Access to PCR tests for HCV and therapy has been inconsistent, with treated patients residing mostly (74%) in the South and Southeast Regions of Brazil. The numbers have dropped since March, possibly due to the impacts derived from the COVID-19 pandemic, although it should not be accepted as justification.

With that in mind and in line with the national strategy for the micro-elimination of HCV, the Brazilian Society of Nephrology, the Brazilian Society of Hepatology, and the Brazilian Liver Institute united to create the “Brazilian Registry for the Elimination of Hepatitis C in Dialysis Units”. The purpose of the project is to identify patients with HCV on dialysis in Brazil, offer treatment, monitor, and record cases of virologic cure. The initiative requires the establishment of an effective, viable, affordable care workflow with adjustments to support regional specificities. This national initiative requires the coordinated efforts of national and regional leadership in nephrology and hepatology and the support of public administration.

Calls for unity have been heard among Brazilian nephrologists for years. This project offers nephrologists a chance to unite around a significant purpose. Dialysis units and nephrologists from all corners of Brazil may now rally for the benefit of the reason why this - or any - medical specialty exists: our patients.
